# Supplementary material for: Exposure to 16 Hz Pulsed Electromagnetic Fields Protect the Structural Integrity of Primary Cilia and Associated TGF-β Signaling in Osteoprogenitor Cells Harmed by Cigarette Smoke
Source: Int J Mol Sci. 2021 Jun 29;22(13):7036. doi: 10.3390/ijms22137036 (PMC8268780; doi:10.3390/ijms22137036)
Supplement: Supplementary file 1 [file ijms-22-07036-s001.zip › ijms-1255723-SI.pdf]

International Journal of
Molecular Sciences

MDPI

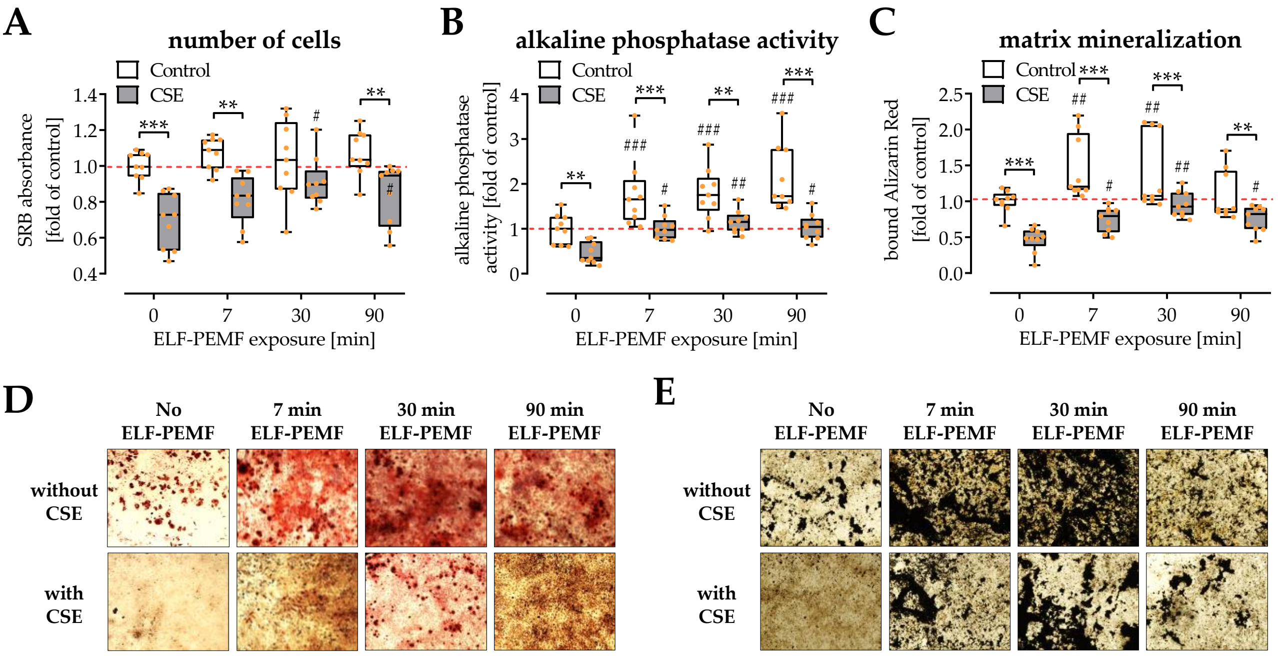

Supplementary Figure S1: Osteogenic differentiation of SCP-1 cells is impaired by exposure to 5% cigarette smoke extract (CSE) and partly rescued by daily exposure to 16 Hz ELF-PEMFs. SCP-1 cells were osteogenically differentiated for upt o 21 days with 7, 30 or 90 min daily exposure to 16 Hz ELF-PEMFs. (A) Sulforhodamine B (SRB) staining was used to quantify cell numbers by total protein content after 21 days. (B) As early osteogenic marker alkaline phosphatase activity was determined after 14 days of diferentiation. (C) Formation of mineralized matrix was quantified by Alizarin Red staining. Mineralized Matrix was additionally visualized by (D) Alizaring Red and € von Kossa staining. N = 3, n = 3. Data are presented as box plots (Min to Max with single data points). Assays were performed as previouly published [Ehnert S. et al. Bone Rep 2015]. Data were compared by non-parametric two-way ANOVA followed by Tukey's multiple comparison test: ** $p < 0.01$ and *** $p < 0.001$ as indicated; $^{\#}$ $p < 0.05$, $^{\#\#}$ $p < 0.01$, and $^{\#\#\#}$ $p < 0.001$ as compared to the respective control (no ELF-PEMF).
